# Exploring Nano-Delivery Systems to Enhance the Edaravone Performance in Amyotrophic Lateral Sclerosis Treatment

**DOI:** 10.3390/ijms26052146

**Published:** 2025-02-27

**Authors:** Brandon Aguiar, Ana Rita Alfenim, Cláudia Sofia Machado, Joana Moreira, Miguel Pinto, Francisco J. Otero-Espinar, Fernanda Borges, Carlos Fernandes

**Affiliations:** 1 CIQUP-IMS, Department of Chemistry and Biochemistry, Faculty of Sciences, University of Porto, R.Campo Alegre s/n, 4169-007 Porto, Portugal; brandonlageaguiar@gmail.com (B.A.);; 2 Associate Laboratory i4HB—Institute for Health and Bioeconomy, Faculty of Pharmacy, University of Porto, R. Jorge de Viterbo Ferreira 228, 4050-313 Porto, Portugal; 3 UCIBIO—Applied Molecular Biosciences Unit, Laboratory of Toxicology, Department of Biological Sciences, Faculty of Pharmacy, University of Porto, R. Jorge de Viterbo Ferreira 228, 4050-313 Porto, Portugal; 4 Pharmacology, Pharmacy and Pharmaceutical Technology Department, Faculty of Pharmacy, Insitute of Materials (iMATUS), University of Santiago de Compostela (USC), 15782 Santiago de Compostela, Spain; 5 Health Research Institute of Santiago (IDIS), 15706 Santiago de Compostela, Spain

**Keywords:** edaravone, amyotrophic lateral sclerosis, hybrid nanoparticles, nanostructured lipid carriers

## Abstract

Edaravone is one of the treatment options for Amyotrophic Lateral Sclerosis, but its therapeutic efficacy is limited due to the incapacity to cross the blood–brain barrier, as well as its short life span and poor stability, which is ultimately caused by its tautomerism in physiological condions. This work presents an overview about the use of several nanoformulations based on polymeric, protein, lipidic, or hybrid structure as suitable and stable drug delivery systems for encapsulating edaravone. We also evaluated the functionalization of nanoparticles with pegylated chains using the polyethylene glycol or tocopherol polyethylene glycol succinate and the possibility of preparing polymeric nanoparticles at different pH (7.4, 9, and 11). Edaravone was sucessfully encapsulated in polymeric, lipid–polymer hybrid, and lipidic nanoparticles. The use of higher pH values in the synthesis of polymeric nanoparticles has led to a decrease in nanoparticle size and an increase in the percentage of encapsulation efficiency. However, the resulting nanoformulations are not stable. Only polymeric and hybrid nanoparticles showed good stability over 80 days of storage, mainly at 4 °C. Overall, the nanoformulations tested did not show cytotoxicity in the SH-SY5Y cell line except the nanostructured lipid carrier formulations that showed some cytotoxicity possibly due to lipidic peroxidation. In conclusion, this work shows that edaravone can be encapsulated in different nanocarriers that could act as an interesting alternative for the treatment of Amyotrophic Lateral Sclerosis.

## 1. Introduction

Amyotrophic Lateral Sclerosis (ALS) is a relentlessly progressive neurodegenerative disorder affecting the motor pathways, characterized by the progressive degeneration of motor neurons and muscle atrophy, resulting in paralysis and respiratory failure, leading to death within 2 to 5 years after symptom onset [1,2].

At present, treatment options are limited to symptom management and respiratory support, and the only approved medications are Riluzole, edaravone (EDV) and sodium phenylbutyrate/taururosodiol (a combination of two drugs), which only mildly alleviate symptoms and extend life expectancy in some patients [3]. Edaravone was first approved in Japan in 2001 for the treatment of stroke [4]. Although the mechanisms of action are not yet fully understood, both in vitro and in vivo studies suggest that EDV, by scavenging free radicals, may attenuate neuroinflammation, by providing protection against oxidative stress [5]. However, EDV presents some limitations such as the incapacity to effectively cross the blood–brain barrier (BBB), its short life span, poor stability, and solubility, and the need for repeated high-dose injections, twice a day, for patients [6]. With this in mind, the development of more efficient targeted drug delivery options remains an urgent task for the improvement in the clinical applications of EDV in the treatment of ALS.

In recent years, nanotechnology has allowed the development of new drug delivery approaches that seem promising for the improvement in the bioavailability, pharmacokinetics, and pharmacodynamics of drugs, as well as reducing their side effects [7,8,9,10]. Among the most studied controlled drug delivery systems, polymeric nanoparticles (PNPs), due to their high drug-loading capacity, biodegradability, easy elimination, and biocompatibility, are the most commonly used [11]. Within PNPs, the most commonly used polymers are Poly(Lactic-co-Glycolic acid) (PLGA), which was approved by the Food and Drug Administration (FDA) in January 1989 [12], and Polyethylene glycol [13], which can be combined to form an amphiphilic block copolymer with improved stealth properties and shelf stability [14]. In fact, the co-polymer PLGA-PEG was already used to prepare micelles and encapsulate EDV to improve its therapeutic capacity against ischemia [15]. Through the method of solvent evaporation, the authors synthesized stable EDV-loaded micelles with sizes between 120 and 250 nm. Aside from PNPs, lipid nanoparticles have been widely used as drug delivery systems, owing to their lipid biocompatibility and versatility [16], within which nanostructured lipid carriers (NLCs), lipid nanoparticles characterized by a solid lipid core, consisting of a mixture of solid and liquid lipids, have proved to be very promising [17]. A new category of nanoparticles has also emerged to combine the benefits of both polymeric and lipid nanoparticles giving rise to the lipid–polymer hybrid nanoparticles (LPHNPs) [18], characterized by a polymeric core, an inner lipid layer and an outer lipid–PEG layer [19].

In this pioneering study, we aimed to explore and optimize the encapsulation of EDV inside different types of NPs and examine further applications of the resulting nanoformulations in in vitro models of ALS. For that, PNPs, NLCs, and LPHNPs were synthesized, and the impact of different EDV/NP backbone ratios, backbones, and pH was evaluated. The resulting nanoformulations were fully characterized as possible drug delivery systems to overcome the limitations of the use of EDV as a treatment for ALS, through the evaluation of their hydrodynamic size (D*_DLS_*), polydispersity index (PDI), zeta potential, and encapsulation efficiency (EE%), as well as their cell viability profile in a neuroblastoma (SH-SY5Y) cell line.

## 2. Results and Discussion

### 2.1. Determination of Physicochemical Properties of Edaravone

**Figure 1:**
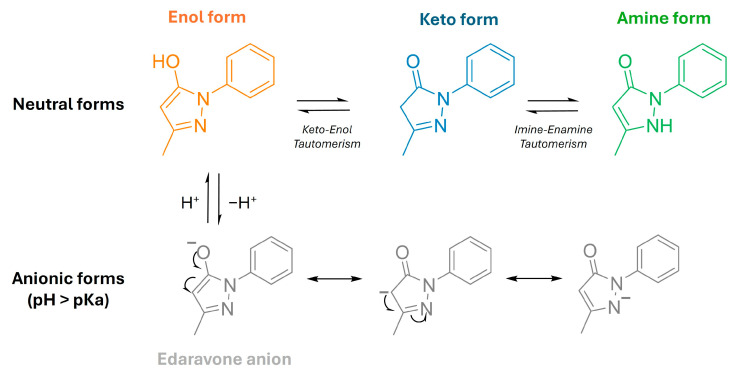
Schematic representation of the tautomerism of edaravone.

Edaravone (3-methyl-1-phenyl-2-pyrazolin-5-one, EDV) is an antioxidant operating by a one-electron transfer mechanism as a scavenger of peroxyl radicals and peroxynitrite, among others [20]. Edaravone exhibits keto–enol and imine–enamine tautomerisms and has three isomeric structures that have different physical and chemical properties (Figure 1): the keto/imine; the enol, with a hydroxy group at the 5-position (phenol-like structure); and the enamine, with a hydrogen atom on the nitrogen atom at the 2-position. Edaravone has weak acid properties, as the acid dissociation constant (pKa) is 7.0, and can dissociate to afford the anion form, which has three resonance structures. Thus, under physiological conditions (in water, pH 7.4), EDV is found as a mixture of neutral and anionic forms [21].

In fact, previous studies showed that, in non-polar solvents, the keto-form of EDV is the most stable one, while the enol form and EDV anion are more prevalent in water [22]. This feature diminishes its ability to cross the blood–brain barrier (BBB) [20]. Moreover, it can be extruded from the brain since it is a substrate of the P-glycoprotein efflux pump [23]. These data are associated with its very short half-circulation time (0.2–5 h), poor oral bioavailability, poor aqueous solubility and stability, and rapid metabolism, which requires EDV to be intravenously administrated twice a day (over 30 min of administration) for a period of 14 days, which is considered a huge burden for patients [24].

Keeping in mind our goal of exploring the encapsulation of EDV in nano-delivery systems, the first step of our studies was to implement an analytical chromatographic method by UHPLC (see experiments). The analytical method was used for both the determination of relevant EDV physicochemical properties and its quantification.

Due to its instability and unpredictable ADME transport properties in in vivo models, biomimetic chromatographic techniques were used to estimate the lipophilicity, intracellular uptake, and body distribution. It was described that octanol/water partition coefficients are not suitable for the estimation of the in vivo distribution of molecules, especially when they are charged at a physiological pH value [25]. So, we estimated the CHI log D values of EDV at two different pH values (2.6 and 7.4), which showed that EDV, under acidic conditions, had a more lipophilic profile (CHI logD_2.6_ = 1.007, Table S1, Supporting Information) when compared with its form under physiological conditions (CHI logD_7.4_ = 0.754, Table S1, Supporting Information). This mainly occurs because, at neutral pH, EDV is in its anionic form, decreasing its affinity to the reverse phase column. Furthermore, comparing the CHI logD_2.6_ of EDV with the data obtained by our group with tolcapone (CHI logD_2.6_ = 2.547) [26], a well-known BBB permeable drug, it is possible to justify that the hydrophilic profile of EDV in physiological conditions is one of the main causes that enable its cross through BBB even with the recognition of efflux pumps. By using the HPLC column functionalized with phospholipids, the value of cell partition (Kpcell) of 0.20 was obtained, which means that under physiological conditions, most of EDV is found outside of the cell [27]. These data also demonstrate the inability of EDV to enter inside the cytoplasmatic matrix of the cells.

We also determined the solubility of EDV in water and in three different lipidic co-solvents (Transcutol HP, CapryolTM 90, and CapryolTM PGMC). The choice of one of these co-solvents is crucial for the synthesis of lipid-polymer hybrid NPs (LPHNPs) and nanostructured lipid carriers (NLC). The lipids were chosen based on what is known for EDV in the literature [23]. Edaravone was left under orbital stirring for 24 h at 25 °C. The data presented in Table S1 (Supporting Information) demonstrate the higher solubility of EDV in Transcutol HP when compared with water, which suggests that even in anionic form, this drug has a greater tendency to be soluble in apolar solvents than in water. With this in mind, we chose to use nanoparticles with a hydrophobic core. Selecting a suitable liquid lipid is a critical step for the preparation of LPHNPs and NLCs.

Next, we explored different nanomaterial types to attain a satisfactory entrapment of EDV. For this, we studied the effect of the (i) ratio of EDV and polymer content (10, 20, and 30%, *w*/*w*), (ii) the pH (7.4, 9, and 11) and the (iii) type of NP backbone (polymeric, albumin, lipidic, and hybrid lipidic–polymeric-based composition), as our final goal is the delivery of the most promising nanoformulations into the brain. For this, we aimed for NPs with a hydrodynamic diameter (D*_DLS_*) between 100 and 300 nm, which is described as optimal for brain delivery nanosystems [28], a polydispersity index (PDI) lower than 0.2, which translates into a very monodispersed system [29,30], and negative zeta potential (ZP) values above 30 mV were also required to have a higher electrostatic repulsion, preventing nanoparticles from aggregating, thus increasing their stability. Also, negative ZP is usually considered advantageous for drug delivery systems since it prevents non-specific interactions with blood components, reducing the risk of immunization and clearance by the immune system [31,32].

### 2.2. Synthesis and Characterization of Polymeric- and Albumin-Based Nanoparticles

A first attempt to encapsulate EDV was performed using PLGA, a polymer widely used in drug delivery systems. Formerly, our group demonstrated that PLGA NPs act as promising carriers for drugs through the intestinal barrier and BBB [33,34,35]. Additionally, the PLGA NPs were also dopped with 10% (*w*/*w* of PLGA) of PEG to evaluate its effect on the stabilization of NPs and EDV encapsulation, since the presence of the amphiphilic profile of PEG chains can enhance the entrapment of the drug. PLGA and PLGA-PEG NPs loaded with EDV were labelled as PLGA@EDV and PLGA-PEG@EDV, respectively.

Furthermore, ANPs were also used as carriers for EDV since it is described that this type of carrier has shown advantageous characteristics, including biodegradability, biocompatibility, and favourable toxicological profiles. Also, they can specifically bind to the albumin receptors (glycoprotein 60 and SPARC), increasing the cellular uptake and BBB permeability of NPs [36]. Albumin NPs can transport hydrophobic, hydrophilic, and lipophilic molecules, which looks like a suitable strategy for entrapping EDV, a drug that presents tautomerism in physiological conditions [22]. Albumin NPs loaded with EDV were labelled as ANP@EDV.

**Figure 2:**
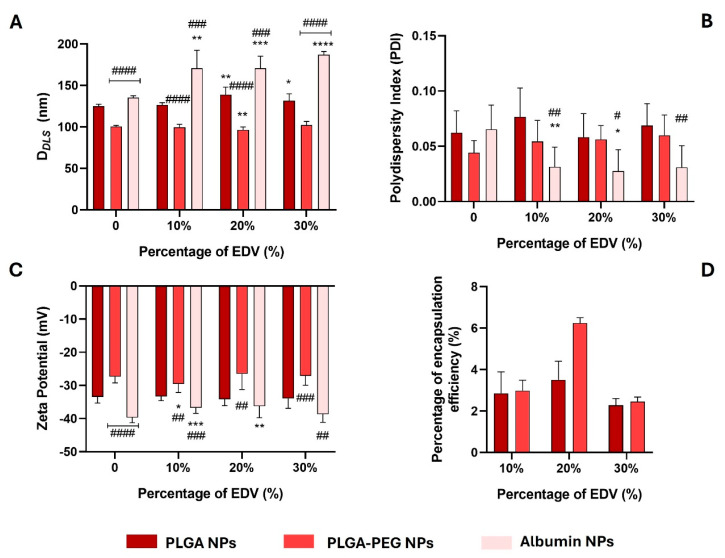
Measurement of hydrodynamic average sizes ((**A**), D*_DLS_*), polydispersity index ((**B**), PdI), and zeta potential ((**C**), ZP) of PNPs and ANPs with and without EDV (10%, 20%, and 30% *w*/*w* polymer. The percentage of encapsulation efficiency ((**D**), EE%) was determined using UHPLC technique. Values are presented as mean ± standard deviation (SD) of at least three independent syntheses of each nanoformulation. Statistical comparisons were made using two-way ANOVA. In all cases, *p* values lower than 0.05 were considered significant (* *p* < 0.05, ** *p* < 0.01, *** *p* < 0.001, **** *p* < 0.0001 comparing unloaded and loaded nanoformulations; # *p* < 0.05, ## *p* < 0.01, ### *p* < 0.001, #### *p* < 0.0001 comparing with data of PLGA nanoparticles loaded with EDV for the same initial percentage of drug used).

The synthesis of PNPs was performed via the nanoprecipitation method, while ANPs were prepared by the desolvation method using glutaraldehyde as a cross-linking reagent. For both methods, loaded nanoformulations were prepared using 10, 20, and 30% (*w*/*w* of total polymer or albumin) of EDV. All nanoformulations were characterized for their hydrodynamic diameter (D*_DLS_*), polydispersity index (PDI), and zeta potential (ZP) immediately after synthesis. The results are presented in Figure 2.

By measuring the D*_DLS_*, it was observed that the encapsulation of EDV at different percentages of initial feeding (10, 20, and 30%) caused a significant change in NP size, mainly in the case of ANPs (all conditions tested) and PLGA-based NPs (using 20 and 30% of initial EDV feed) (Figure 2A). Empty ANPs showed a D*_DLS_* value of 135.5 ± 2.1 nm, while all the loaded albumin NPs had sizes larger than 170 nm (Figure 2A). Also, both PLGA-PEG@EDV and ANPs@EDV NPs showed significant data differences when compared with PLGA@EDV NPs for the same EDV feed (Figure 2A). Thus, EDV-loaded albumin NPs presented D*_DLS_* values larger than PLGA-based NPs (D*_DLS_* = 127–139 nm). In contrast, PLGA-PEG@EDV NPs present sizes in the range of 96 nm to 102 nm, similar to those previously described by our group as suitable for permeation in the in vitro model of BBB [33].

All the nanoformulations (Figure 2B) presented a monodisperse profile with PDI values lower than 0.1. After being loaded with EDV, albumin NPs showed lower polydispersity when compared with empty ones, and a significant difference when compared with PLGA NPs for the same initial EDV feed (Figure 2B). This may possibly occur due to a stabilization of the albumin NPs in the presence of the EDV. In terms of surface charge, all nanoformulations ensured a highly negative ZP value (ZP > −26.5 mV), since PLGA has a carboxylic acid at the end of its polymeric chain, and under physiological conditions, it can be deprotonated [34]. Albumin NPs are negatively charged at physiological pH since albumin-measured isoelectric point is around ~4 [37]. The encapsulation of EDV in ANPs (10 and 20% of initial feed) and PLGA-PEG NPs (10% of initial feed) led to a significant difference in ZP values when compared with empty related NPs, causing a decrease and an increase in ZP, respectively (Figure 2C). The decrease in ZP value in ANP@EDV NPs (10 and 20%) could be correlated with the data observed in the PDI values (Figure 2B), which suggests that EDV can have a role in the modification of the structure of albumin NPs. Moreover, by comparing the data of PLGA-PEG NPs (ZP = −26.5 and −29.5 mV) with PLGA NPs (ZP = −33.2 and −34.2 mV), it is possible to verify a decrease in the absolute value of ZP, which can be related to the insertion of the PEG chains that increases the neutral profile of the NPs.

No significative changes were observed in each nanoformulation in terms of D*_DLS_*, PDI, and ZP values, taking into account the different initial EDV feed used (Figure 2A–C). In terms of encapsulation efficiency (EE%), significant differences were observed for the different conditions tested. It is worth noting that it was not possible to determine the amount of EDV inside albumin NPs, since, despite the several methods of elution tested and purification methods (centrifugation and lyophilization), the chromatogram peak of the drug was not detected isolated due to the presence of the peak of glutaraldehyde. We suspected that, somehow, the cross-linking reagent reacted with EDV and conjugated it with the albumin core of NPs, and, because of that, it was impossible to further study this nanoformulation. Otherwise, by correlation with the calibration curve (Figure S1, Supporting Information), the EE% was determined for the nanoformulations based on PLGA and PLGA-PEG prepared with different initial EDV feeds (Figure 2D). For all conditions tested, PLGA-PEG NPs showed a higher capacity of entrapping the EDV when compared with PLGA NPs, due to the amphiphilic profile of the PEG that could interact with the drug, increasing its affinity for the nanoparticle. This fact was noticed mainly when EDV was used in the initial percentage of 20%. In this case, PLGA-PEG NPs were able to entrap around 6.3% of the initial EDV used in the synthesis, which corresponds to a final concentration of the drug of 180.2 µM.

As the best results were obtained for EDV-loaded PLGA and PLGA-PEG NPs prepared with 20% of initial drug feed, these nanoformulations were selected to proceed with the studies of stability and cytotoxicity.

### 2.3. Effect of pH in the Synthesis of PLGA and PLGA-PEG Nanoparticles

Due to the EDV tautomerism presented at different pHs, we also evaluated the effect of basic conditions (pH 9 and 11) on its encapsulation efficiency on PLGA and PLGA-PEG NPs. Acidic conditions were not tested due to the cleavage of the PLGA chains occurring through the hydrolysis of the ester groups [33]. Nevertheless, since the anionic form of EDV is the one that was related to EDV antioxidant activity, it is more relevant to encapsulate this bioactive species.

**Figure 3:**
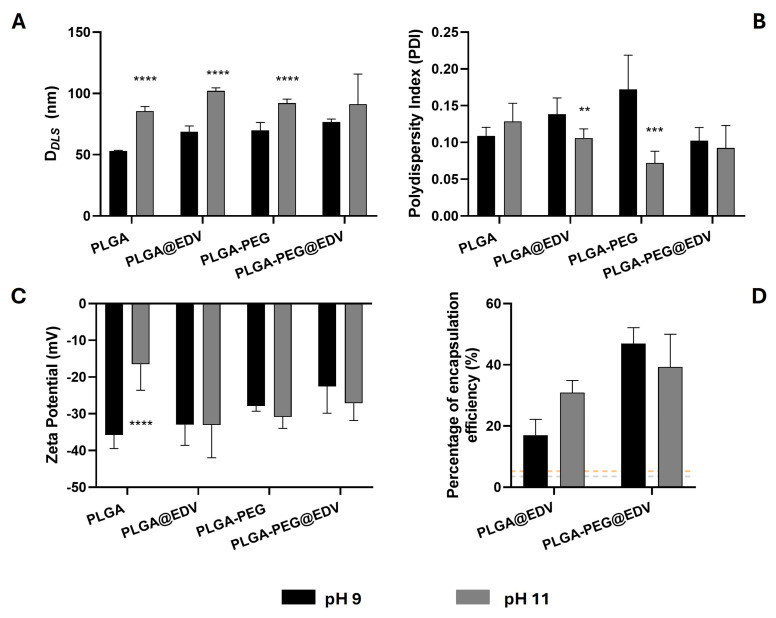
Evaluation of pH effect on size, surface charge, and EE% of PLGA and PLGA-PEG NPs. D*_DLS_* (**A**), PdI (**B**), and ZP values (**C**) of PLGA-based NPs with and without EDV (20%, *w*/*w* polymer). The percentage of encapsulation efficiency ((**D**), EE%) was determined using UHPLC technique. Values are presented as mean ± SD of at least three independent syntheses of each nanoformulation. Statistical comparisons were made using two-way ANOVA. In all cases, *p* values lower than 0.05 were considered significant (** *p* < 0.01, *** *p* < 0.001, **** *p* < 0.0001 comparing the data between the two pH values).

Considering the data obtained previously, the initial drug feeding of 20% was selected. The morphology and EDV content of NPs was evaluated (Figure 3). All nanoformulations prepared under basic conditions presented a lower D*_DLS_* when compared with those prepared with water and 20% of initial EDV feed (Figure 3A, D*_DLS_* of PLGA@EDV and PLGA-PEG@EDV = 126.6 nm and 99.4 nm, respectively). As far as we know, the use of basic conditions to synthesize PLGA NPs was never reported. The significant decrease in NP size can be ascribed to a pH effect in the nucleation and growth process during the formation of NPs, maybe due to the deprotonation of the carboxylic groups of PLGA [38], which could promote the earlier stabilization of NPs, avoiding the growth process of particles. Comparing the PLGA and PLGA-PEG NPs, a tendency for the non-functionalized nanoformulations to be in smaller sizes was observed, which could corroborate the proposed hypothesis.

A high polydisperse profile of the PLGA@EDV and PLGA-PEG@EDV NPs synthesized with a basic aqueous solution (Figure 3B, PDI > 0.07) was noticed. A tendency for higher values of PDI, namely for NPs prepared with pH 9 than pH 11, as well as for PLGA NPs, was also observed when compared with PLGA-PEG NPs. Once more, the higher basic strength of the aqueous phase caused a higher deprotonation of the carboxylic groups, which led to a faster stabilization of the nuclei, causing their smaller sizes. Otherwise, this effect was ameliorated with the presence of a PEG chain that encompasses the faster deprotonation of the PLGA, allowing a longer nucleation and growth process. The basic pH did not significantly affect the surface charge of NPs when compared with those prepared with ultrapure water (Figure 3C). As shown in Figure 3D, a significant increase in the EE% from 3.5–6.3% to 17.0–46.9% with the replacement of ultrapure water by basic aqueous solution.

### 2.4. Synthesis and Characterization of Lipid–Polymer Hybrid Nanoparticles and Nanostructured Lipid Carrier

Since EDV showed superior solubility in lipidic-based co-solvents (Table S3, Supporting Information) than in water, LPHNPs and NLC were prepared by the nanoprecipitation and single emulsion methods, respectively, using Transcutol HP [39], which was the lipidic co-solvent that presented the highest solubility for the drug (Table S3, Supporting Information). Hybrid NPs were prepared by maintaining the PLGA or PLGA-PEG as the shell and adding d-α-tocopheryl polyethylene glycol 1000 succinate (TPGS). This compound has an amphiphilic nature and is described as an emulsifier, gelling agent, solubilizer, and dispersant agent [40]. Moreover, TPGS has been used for other neurodegenerative therapies, acting as a trap of free radicals and interrupting damaging cell chain reactions [41]. Under this work, LPHNPs loaded with EDV were labelled as Hyb-PLGA@EDV, Hyb-PLGA-PEG@EDV, and Hyb-PLGA-TPGS@EDV, depending on the type of functionalization used.

**Figure 4:**
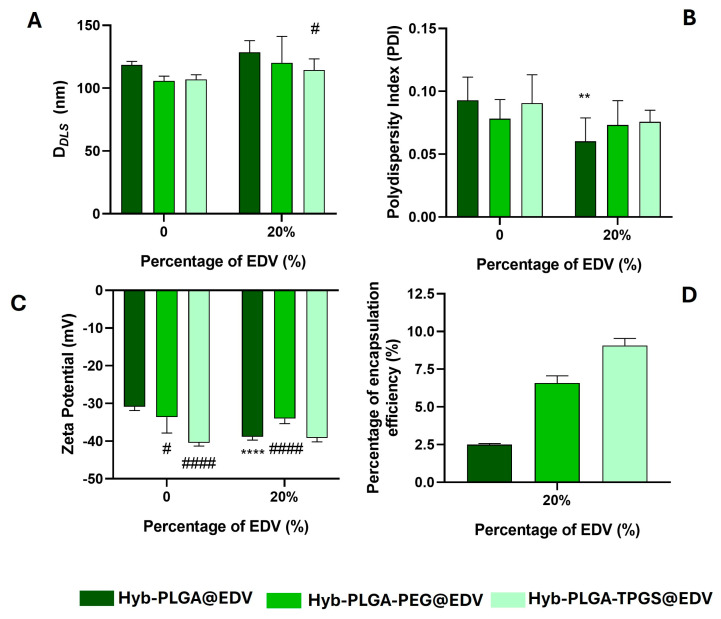
Measurement of D*_DLS_* (**A**), PdI (**B**), and ZP values (**C**) of LPHNPs with and without EDV (20%, *w*/*w* polymer). The percentage of encapsulation efficiency ((**D**), EE%) was determined using UHPLC technique. Values are presented as mean ± SD of at least three independent syntheses of each nanoformulation. Statistical comparisons were made using two-way ANOVA. In all cases, *p* values lower than 0.05 were considered significant (** *p* < 0.01, **** *p* < 0.0001 comparing unloaded and loaded nanoformulations; # *p* < 0.05, #### *p* < 0.0001 comparing with data of Hyb-PLGA nanoparticles loaded with EDV for the same initial percentage of drug used).

As observed in Figure 4, the encapsulation of EDV caused a slight increase in size and a decrease in polydispersity of hybrid NPs when compared with the empty ones while the surface charge remained unaltered (Figure 4A–C), with the exception of Hyb-PLGA@EDV NPs. The surface charge of hybrid NPs presented higher negative values (ZP = −34 to −39 mV) when compared with polymeric NPs (ZP = −34.2 and −26.5 mV for PLGA and PLGA-PEG@EDV NPs, respectively), which proved the presence of the carboxylic acid groups of PLGA at NP’s surface. It is suggested that the hybrid NPs have a monolithic architecture: a lipidic shell with a polymeric coating where the drug is entrapped [42]. The higher values of ZP after encapsulation in the case of Hyb-PLGA@EDV NPs can be ascribed to the presence of an anionic form of EDV on the surface of NPs. With the functionalization of the NP surface with PEG and TPGS chains, a slight decrease in size was observed (Figure 4A), while a relevant difference in ZP was detected (Figure 4C), mainly for the Hyb-PLGA-PEG@EDV NPs. Not only did the functionalization with PEG or TPGS affect the amount of EDV entrapped in the hybrid NPs (Figure 4D), but also the type of PEGylated chain, since NPs functionalized with TPGS showed to have an EE% of about 4-fold times higher than unfunctionalized NPs (Figure 4D).

**Figure 5:**
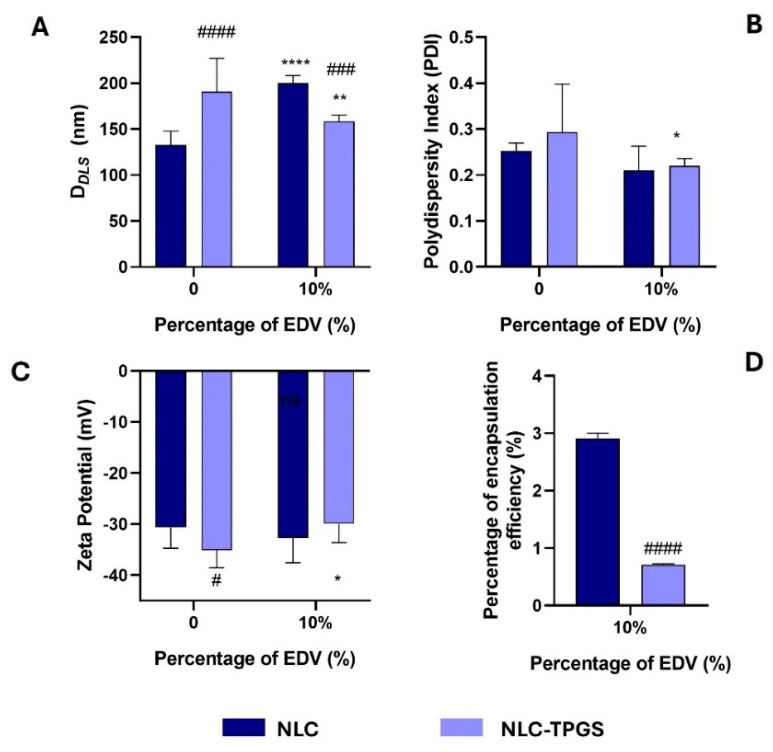
Determination of D*_DLS_* (**A**), PdI (**B**), and ZP values (**C**) of NLC with and without EDV (10%, *w*/*w* lipid). The percentage of encapsulation efficiency ((**D**), EE%) was determined using UHPLC technique. Values are presented as mean ± SD of at least three independent syntheses of each nanoformulation. Statistical comparisons were made using two-way ANOVA. In all cases, *p* values lower than 0.05 were considered significant (* *p* < 0.05, ** *p* < 0.01, **** *p* < 0.0001 comparing unloaded and loaded nanoformulations; # *p* < 0.05, ### *p* < 0.001, #### *p* < 0.0001 comparing with data of NLC loaded with EDV for the same initial percentage of drug used).

The method of single emulsion was used to prepare EDV-loaded NLC. In a first attempt, the same percentage of initial EDV feeding (20%, *w*/*w* of lipid) was tested, but after 24 h of storage, it was possible to observe the precipitation of the components of the nanoformulation. This occurred probably due to the difference between the NPs presented above and NLC regarding the composition and structure. Because of that, a preliminary evaluation of the optimal initial feeding was performed using the initial percentages of 5, 10, and 15% of EDV, being determined that, for NLC, higher values of EE% were obtained for the 10% (Figure S2, Supporting Information). Considering this parameter, we prepared TPGS-functionalized NLC loaded with EDV by the same method reported above. NLC and NLC-TPGS loaded with EDV were labelled as NLC@EDV and NLC-TPGS@EDV, respectively, and the data of their morphological characterization are presented in Figure 5.

After EDV encapsulation, and when comparing with empty NLC, a difference in D*_DLS_* and PDI values for both NLC prepared (Figure 5A), as well as in ZP values for NLC-TPGS@EDV (Figure 5B,C), was observed. The drug encapsulation caused an increase in NLC size (from 132.8 nm to 200.1 nm), while a decrease was observed for NLC-TPGS@EDV (from 190.8 to 158.7 nm) (Figure 5A). Furthermore, it led to a decrease in PDI (from 0.253 to 0.210 and 0.294 to 0.220 for NLC@EDV and NLC-TPGS@EDV, respectively) (Figure 5B) for both NLC prepared and of ZP (from −35.1 to −29.9 mV) for NLC-TPGS@EDV (Figure 5C). The presence of TPGS in the nanoformulation increased the size of the NPs in the absence of EDV, while the opposite data were obtained in the presence of the drug (Figure 5A). The presence of EDV may have stabilized the formation of smaller emulsion drops that gave rise to the NLC. Despite that, the encapsulation of EDV was more efficient using NLC without TPGS functionalization (Figure 5B).

### 2.5. Stability Study of PNPs, LPHNPs, and NLC

**Figure 6:**
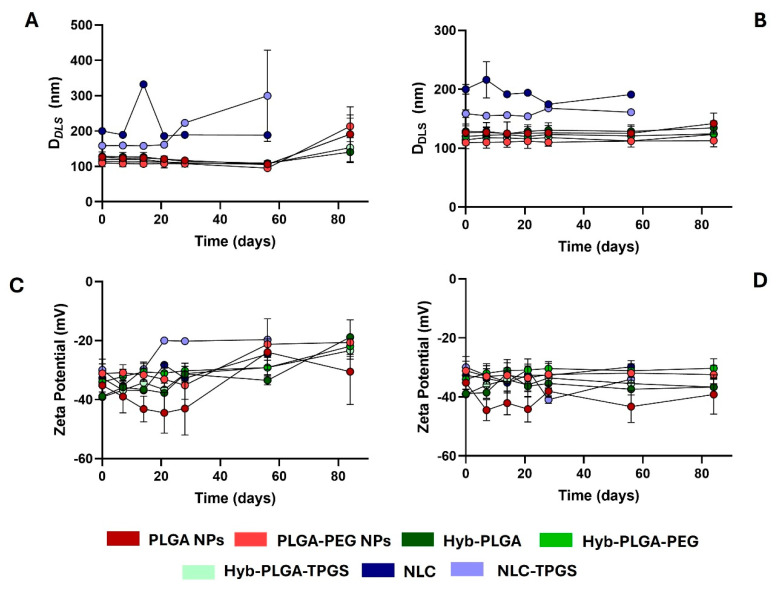
Measurement of D*_DLS_* (**A**,**B**) and ZP values (**C**,**D**) of PNPs, LPHNP hybrid, and NLC loaded with EDV at different timepoints (days) under storage conditions at 25 °C (**A**,**C**) and 4 °C (**B**,**D**). Values are presented as mean ± SD of at least three independent experiments.

The storage stability of the nanoformulations prepared under this work, with exception of albumin NPs and PLGA-based NPs prepared under basic conditions, was evaluated for 6 weeks at 4 and 25 °C, by the evaluation of morphology change over time. The analysis of D*_DLS_* and ZP in function of time is presented in Figure 6, while the PDI values are presented in Figure S3 (Supporting Information).

Despite the promising data, the stability of loaded nanoparticles prepared under basic conditions was compromised by the presence of a precipitate and the appearance of a pink colour after 1 week of storage, which could be attributed to the degradation of EDV and, subsequently, the polymerization of the EDV trimer, invalidating the use of these nanoformulations in the following studies [22]. Although not suitable for EDV, the use of basic aqueous solution could be a novel approach to fine-tune the morphology and physicochemical features of PLGA NPs.

The same precipitation occurred for NLC, but only after 60 days of the experiment. Until the destabilization process occurred, it was observed that the sizes of NLC and NLC-TPGS were more stable when NPs were stored at 4 °C than at 25 °C (Figure 6A,B). This observation was mainly detected for the NLC-TPGS nanoformulation, which had its D*_DLS_* increased from 158.7 to 299.8 nm after 56 days of storage at 25 °C (Figure 6A). This modification was encompassed by a decrease in ZP value from −29.9 to −19.6 mV (Figure 6C) and an increase in PDI values from 0.2 to 0.4 (Figure S3, Supporting Information). We suggest that this decrease in ZP value had an important role in the precipitation process of the NPs, as already described by Russo et al. [43].

Similar data were obtained for PLGA-based NPs after 84 days of experiment stored at 25 °C (Figure 6A,C and Figure S3, Supporting Information). For polymeric NPs, the size increased by about 51.4% and 95.9% for PLGA@EDV and PLGA-PEG@EDV, respectively, while, for the hybrid NPs, it increased by about 9.3%, 59.2%, and 33.6% for Hyb-PLGA@EDV, Hyb-PLGA-PEG@EDV, and Hyb-PLGA-TPGS@EDV NPs, respectively. Despite that, for both types of NPs, the size was lower than 200 nm at the end of the experiment. In general, the storage of polymeric and hybrid NPs at 4 °C maintained their initial features, even with the inherent issues related to EDV stability, which highlights the high performance of both polymeric and hybrid nanoparticles to overcome the limitations of this drug.

### 2.6. Evaluation of Cytotoxic and Antioxidant Profile of Nanoformulations

**Figure 7:**
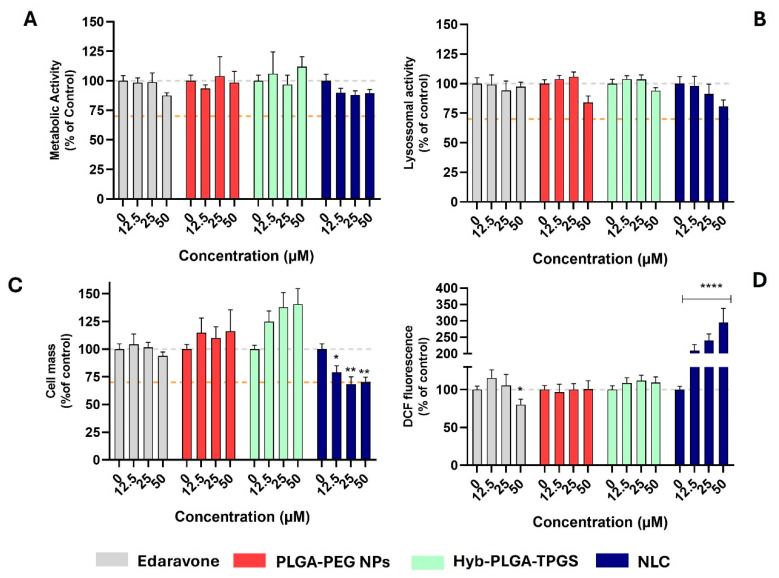
Cytotoxic effects of EDV and PLGA@EDV, Hyb-PLGA-TPGS@EDV, and NLC@EDV in SH-SY5Y cells (12.5, 25, and 50 μM) after 24 h of exposure, by measuring the metabolic activity (**A**), lysosomal activity (**B**), and cell mass (**C**) by the resazurin reduction method, neutral red uptake, and SRB assays, respectively. Intracellular ROS levels after exposure of SH-SY5Y cells (**D**) were also measured after 24 h of exposure with EDV and nanoformulations. The data are expressed as the means of at least four independent experiments together with the standard error mean (mean ± SEM). Statistical comparisons were made using two-way ANOVA. In all cases, *p* values lower than 0.05 were considered significant (* *p* < 0.05, ** *p* < 0.01, **** *p* < 0.0001 vs. the control data). The grey dot line represents the mean of control cells (100%), and the orange dashed line represents the cell viability limit of 70% (ISO 10993-5, https://www.iso.org/obp/ui/\#iso:std:iso:10993:-5:ed-3:v1:en, accessed on 23 January 2025).

From the data obtained so far, one nanoformulation from each type (polymeric, hybrid, or lipidic) of NPs was chosen. Thus, the cytotoxic effect of PLGA-PEG@EDV, Hyb-PLGA-TPGS@EDV, and NLC@EDV in the cholinergic differentiated SH-SY5Y cell line was assessed by measuring the metabolic and lysosomal activities, as well as cell mass, through resazurin reduction, neutral red uptake, and sulforhodamine B (SRB) assays [35]. The cells were treated with different concentrations of nanoformulations considering the concentration of EDV presented in the nanocarriers (12.5, 25, and 50 µM). EDV was tested as well at the same concentrations for comparison. After 24 h of treatment, the end-points were evaluated, and the obtained data are presented in Figure 7.

As observed in Figure 7A–C, the data obtained are above the 70% threshold for the cell viability [13,34,35], which translates the safety of EDV and tested nanoformulations in terms of metabolic and lysosomal activities of the neuronal cells. Only NLC@EDV showed a decrease in cell mass (SRB absorbance = 68.3–70.2%) for the two highest concentrations tested (Figure 7C). Despite that no significant differences were detected for control cells, NLC@EDV caused a slight depletion of metabolic activity (resazurin reduction > 88.1%) and lysosomal activity (NR uptake > 80.7%), which was also observed for the empty NLC (Figure S4, Supporting Information). In a previous work from our group, we already reported the inherent cytotoxicity of lipidic-based nanocarriers, which could be attributed to a peroxidation phenomenon in the cells or a lipidic accumulation in the lysosomes [35]. This is in accordance with the results obtained by the measurement of ROS performed by measuring the fluorescence of probe 2,7-dichlorofluorescein (DCF) [26]. Briefly, 2,7-dichlorofluorescein diacetate (DCFH-DA), a nonfluorescent and membrane-permeable probe, enters the cytoplasm, and there, the esterases remove the acetate groups to produce 2,7-dichlorodihydrofluorescein (DCFH), which, because of its polarity, is not cell permeable. The probe DCFH is easily oxidized to DCF, a highly fluorescent compound (excitation 485 nm: emission 530 nm), by several ROS, including hydrogen peroxide, hydroxyl radicals, and nitrogen dioxide [35]. The data presented in Figure 7D showed that both empty (DCF fluorescence = 476.8–1204.8%, Figure S4) and loaded NLC (DCF fluorescence = 209.1–294.5%) caused an increase in ROS production compared with control cells. Interestingly, the increase in ROS production was lower when cells were treated with loaded NLC compared to empty ones, which could be due to scavenging of radicals by the antioxidant activity of EDV.

To corroborate this theory, a preliminary screening of the antioxidant activity of EDV and the nanoformulations tested in cellular studies was performed against ABTS^•+^ radical (Figure S4, Supporting Information) [44]. In this experiment, the EDV (concentration = 300 μM) scavenged the ABTS^•+^ radical in 67.7%, while the loaded nanoformulations only neutralized 10.8 to 45.3% of the radical, with the Hyb-PLGA-TPGS@EDV being the nanoformulation with higher radical scavenging. Meanwhile, the empty nanoformulations showed no capacity to neutralize the radical. With these data, it was possible to corroborate that EDV encapsulated in NLC could decrease the amount of ROS produced by the lipidic structure of the nanocarrier.

## 3. Materials and Methods

### 3.1. Reagents

Acetone and acetonitrile were obtained from Honeywell (Brussels, Belgium). Edaravone (purity > 98%) was purchased from Alfa Aesar (Ward Hill, MA, USA). Poly(lactic-co-Glycolic acid) (PLGA, purity > 99%, molecular weight = 44 kDa) was offered by Corbion (Amsterdam, The Netherlands); methoxy poly(ethylene glycol)-b-poly(lactide-co-glycolide) (mPEG-PLGA, Molecular weight = 5:45 kDA) was acquired from Akina Inc. (West Lafayette, IN, USA). Poloxamer 188 (Kolliphor P188), glutaraldehyde (25% in H_2_O); and human serum albumin and polysorbate 80 (Tween 80) were purchased from Sigma-Aldrich Química SA (Sintra, Portugal). Fetal bovine serum (FBS), non-essential aminoacids (NEAA), penicillin/streptomycin (PenStrep) and trypsin were acquired to PanBiotech (Aidenbach, Germany). Acetic acid (purity for analysis) was obtained from Carlo Erba (Milano, Italy). Tocopherol polyethylene glycol succinate (TPGS, purity > 99%) was acquired from TCI (Zwijndrecht, Belgium), and soy lecithin and glycerol monoesterate 40–55 were kindly provided by Prof. Dr. Fran Otero-Espinar (Department of Pharmacology, Pharmacy and Pharmaceutical Technology, University of Santiago de Compostela, Spain). All liquid lipids (Transcutol HP, Capryol^TM^ 90 and Capryol^TM^ PGMC) were kindly offered by Gattefossé (purity > 98%, Lyon, France). All the used solvents have purity for HPLC analysis. Water was from Milli-Q (Millipore, Burlington, MA, USA).

### 3.2. Studies Using a High-Performance Liquid Chromatography System

The studies of the measurement of the chromatographic hydrophobicity index (CHI) and CHI on Immobilized Artificial Membrane (CHI(IAM)), as well as the quantification of EDV, were performed using a NEXERA-i LC-2040C UHPLC apparatus (Shimadzu, Kyoto, Japan) equipped with a diode array detector and controlled by the LabSolution system (version 5.90 Shimadzu, Japan). The ultra-high-performance liquid chromatography (UHPLC) experiments were performed with a flow rate of 1 mL/min and the temperature set to 40 °C, using an injection volume of 20 μL. In the measurements of CHI and CHI(IAM), the stock solution of EDV in DMSO (10 mM) was diluted in mixtures of the acetonitrile–aqueous (1:1) solution to obtain a final concentration of 250 μM. These mixture solutions containing acetonitrile and aqueous acetic acid 1% (*v*/*v*) or ammonium acetate (30 mM) in ratio of 1:1, with their pH adjusted to 2.6 and 7.4, respectively, by using HCl (1M) or NaOH (1M).

### 3.3. Evaluation of the Chromatographic Hydrophobicity Index

The CHI values at pH 2.6 and 7.4 were determined and calculated using an experimental protocol previously described [26], assessed from the experimental retention times (t_R_) of the EDV and a mixture of reference compounds obtained on the UHPLC system with a Luna C18 (2) column (150 × 4.6 mm, 5 µm, Phenomenex, CA, USA). For the experiments at pH 2.6, mobile phase A was aqueous acetic acid 1% (*v*/*v*), and mobile phase B was acetonitrile. The use of the acidic additive in the mobile phase avoids the ionization of the EDV, improving the chromatographic separation and peak shape. In the case of pH 7.4, the mobile phase A was an aqueous solution of ammonium acetate (30 mM). The following gradient program was applied: 0−6 min 0–100% B, 6−14 min 100% B, 14−16 min 100–0% B. A calibration curve was obtained using a mixture of reference compounds (Table S2, Supporting Information). The results are expressed as mean values of three experiments.

### 3.4. Evaluation of the Chromatographic Hydrophobicity Index on Immobilized Artificial Membrane

The CHI (IAM) values were determined and calculated from the experimental t_R_ of the EDV and a mixture of reference compounds as described previously [45], using a IAM.PC.DD2 column (100 × 4.6 mm, 10 µm, Regis Technologies, Inc., Morton Grove, IL, USA). The mobile phase A was 1% acetic acid solution (pH = 2.6), and the mobile phase B was acetonitrile. The following gradient program was applied: 0−6 min 0–100% B, 6−10 min 100% B, 10−12 min 100–0% B. A calibration curve was obtained using a mixture of compounds (Table S3, Supporting Information). The results are presented as mean values of three experiments.

### 3.5. Quantification of EDV Encapsulated in Nanocarriers

The quantification of EDV was evaluated by using a Luna C18 (2) column (150 × 4.6 mm, 5 µm, Phenomenex, Torrance, CA, USA), as well as a mobile phase based on a mixture of water with 0.1% TFA and methanol (65:35). All solutions were diluted in acetonitrile, filtered through a 0.22 μm pore size filter (Labfil, ALWSCI Corporation, Shaoxing, China), and injected with a volume of 20 μL. The detection was performed at a wavelength of 242 nm, and all data are presented as mean of at least three measurements.

### 3.6. Determination of EDV Solubility in Water and Liquid Lipids

To address the most suitable conditions used in the synthesis, the solubility of EDV was assessed in both water and in different liquid lipids using the shake-flask methodology [46]. For determining water solubility, an excess of EDV (15 mg) was dispersed in 900 µL of Milli-Q water, and the resulting mixture was left under stirring for 24 h at 25 °C in an Eppendorf ThermoMixer^®^ C (Hamburg, Germany) at 600 rpm. After this period, the sample was centrifuged at 10,000 rpm for 10 min, and the supernatant was collected and analyzed in the UHPLC system as described above. For the evaluation of EDV’s solubility in liquid lipids, a total of 3 oils (Transcutol HP, Capryol^TM^ 90 and Capryol^TM^ PGMC) were chosen. Briefly, 10 mg of each oil was placed in a microtube, and an excessive amount of EDV was added. Each sample was vortexed for 30 s and kept at 37 °C in a 600-rpm agitation for 24 h. At the end, all samples were centrifuged at 10,000 rpm for 10 min, at room temperature, and 7 μL of supernatant were weighted and diluted in 1 mL of acetonitrile. The resulting solutions were analyzed as described previously.

### 3.7. Synthesis of EDV-Loaded Nanocarriers

#### 3.7.1. Preparation of EDV-Loaded Polymeric Nanoparticles

The nanoprecipitation method was used to prepare PNPs [33]. Briefly, for EDV-loaded PLGA NPs, EDV (10, 20 or 30%, *w*/*w* of polymer) and PLGA (50 mg) were dissolved in 10 mL pure acetone and added dropwise to a 20 mL aqueous solution (pH 7.4, 9 or 11) containing Kolliphor 188 surfactant (50 mg), under vigorous magnetic stirring. The solutions with different pH (9 and 11) were prepared by adjusting the basicity of the phosphate-buffered solution with NaOH (1 M). The pH was measured before and after the addition of the surfactant in order to maintain the desired basicity. The solution was left under stirring for 45 min, at 25 °C. Acetone was fully evaporated under reduced pressure at 40 °C (80–100 mbar) using a Rotavapor R210 (Büchi, Barcelona, Spain). The nanosuspension was centrifuged (20 min, 13,000 rpm, 4 °C) (Megafuge 16R, Thermo Fisher Scientific, Waltham, MA, USA) and washed thoroughly with cold ultrapure water. The resulting suspension was centrifuged again using the same conditions and the supernatant removed. The resulting pellets were resuspended in 1 mL of water to prepared suspensions of NPs and stored at 4 °C or stored directly in −80 °C to perform the lyophilization process. For PLGA-PEG PNPs, the same method was used as above, with a small change: PLGA (45 mg) was dissolved in 8 mL of acetone and 2 mL of mPEG-PLGA in acetone solution (1.25%, *w*/*v*). Empty NPs were synthesized in the same way except for the addition of EDV.

#### 3.7.2. Preparation of EDV-Loaded Albumin Nanoparticles

For the preparation of the ANPs, 50 mg of albumin was dissolved in 1 mL of ultrapure water, and EDV (10, 20, or 30% *w*/*w* of polymer) was dissolved in 4 mL of absolute ethanol. The organic solution was added dropwise to the aqueous solution, and then 10 µL of glutaraldehyde was added. The solutions were left stirring overnight. The purification of the ANPs was performed following the procedure already described for PNPs. Empty ANPs were synthesized in the same way except for the addition of EDV.

#### 3.7.3. Preparation of EDV-Loaded Lipid–Polymer Hybrid Nanoparticles

For LPHNPs with PLGA (Hyb-PLGA NPs), EDV (20% *w*/*w* of polymer), PLGA (50 mg), and transcutol HP (50 mg) were dissolved in 10 mL of acetone and added dropwise to a 20 mL aqueous solution (ultrapure water) containing Kolliphor 188 surfactant (50 mg). The resulting solution was pre-emulsified using an Ultra Turrax (T18 digital, IKA, Staufen, Germany) at 13,000 rpm for 2 min. After that, the solution was left under magnetic stirring overnight. The preparation of LPHNPs with PLGA-PEG (Hyb-PLGA-PEG NPs) was similar to the process of synthesis of Hyb-PLGA NPs except for the use of 45 mg of PLGA dissolved in 8 mL of acetone and 2 mL of PLGA-PEG in acetone solution (1.25%, *w*/*v*). For the synthesis of LPHNPs with PLGA and TPGS (Hyb-PLGA-TPGS NPs), the same method of Hyb-PLGA NPs synthesis was followed, but with the addition of 5 mg of TPGS. Empty LPHNPs of each type were synthesized in the same way as previously described without the addition of EDV. The purification of the LPHNPs was performed following the procedure already described for PNPs.

#### 3.7.4. Preparation of the EDV-Loaded NLCs

Synthesis and encapsulation of EDV in NLCs was performed using the solvent emulsification/evaporation method [35]. Then, soy lectin (125 mg), glyceryl monostearate (105 mg), liquid lipid (45 mg), and EDV (5, 10 or 15 *w*/*w* % of lipid) were added into a beaker and dissolved in 5 mL of dichloromethane. The mixture was stirred for 10 min to form a homogenous lipid phase. Afterwards, 22.5 mL of the aqueous phase (polysorbate 1%) was added, and an Ultra Turrax (T18 digital, IKA, Staufen, Germany) at 13,000 rpm for 2 min was employed to pre-emulsify the mixture. This pre-emulsion was then sonicated for 1.5 min (10 s ON, 5 s OFF cycles) at 80% amplitude with an ultrasound probe (TS109, Sonoplus, Bandelin, Berlin, Germany) using an ice bath. Finally, the emulsions were vigorously agitated for 4 h to evaporate the solvent and then filtered using an Amicon filter (Merck, Frankfurt, Germany) through centrifugation (50 min, 5000× *g*, 4 °C). The resulting suspension was diluted with 10 mL and centrifuged again. The resulting suspensions were diluted with ultrapure water at a final volume of 15 mL and stored at 4 °C until further use. The same technique was applied to NLCs functionalized with TPGS (NLC-TPGS); however, 55 mg of TPGS was added into the above-referred mixture. Empty NLC were prepared as described above with exception for the addition of EDV.

### 3.8. Lyophilization Process and Storage of EDV-Loaded Nanoparticles

The resulting pellets stored at −80 °C (with at least 24 h of freezing) were lyophilized in a Lyovapor L-200 (Buchi, Barcelona, Spain) using a pressure of 1 mbar. After 48 h of lyophilization, the resulting powder was stored at −20 °C until the chromatographic experiments. Since the lyophilized NPs would not resuspend in water after lyophilized, the samples used on the morphology, stability, and cellular studies were kept under suspension at 4 °C until the beginning of the experiments.

### 3.9. Determination of EDV-Loaded NPs Morphology and Surface Charge

The hydrodynamic size (D*_DLS_*), polydispersity index (PDI), and zeta potential (ZP) distribution of the NPs were determined by dynamic light scattering (DLS) using a Zetasizer Pro (Zetasizer Pro-Blue, Malvern Instruments, Grovewood, UK) equipped with a 4.0 mW internal laser. For the analysis, 100 μL of each nanosuspension was added to 900 μL ultrapure water. All DLS measurements were performed at 25 °C in a DTS1002 cell and at a scattering angle of 90°. The ZP was measured with the same apparatus, using a DTS1070 cell. The experiments were performed in triplicate and under sink conditions.

### 3.10. Stability Study of EDV-Loaded Nanoformulations

The stability of the nanoformulations under storage conditions, at different temperatures (4 °C and room temperature) and over a period of three months were assessed. For this purpose, the nanoformulations were stored under suspension in a fridge (to evaluate the temperature of 4 °C) and in a laboratory shelf without any protection against artificial light. It is worth noting that the experiment was performed between the month 3 and 6, where the average temperature of the laboratory is around 20 °C. At the predetermined period of time, aliquots of the nanoformulations were collected, and the D*_DLS_*, PDI, and ZP of each nanoformulation were measured as described above. The nanoformulations were also observed visually to check for any precipitation and destabilization phenomenon.

### 3.11. Measurement of Encapsulation Efficiency (%) of EDV-Loaded Nanoformulations

The quantification of EDV present in each nanoformulation was performed to evaluate the percentage of encapsulation efficiency (EE%). For the PNPs and LPHNPs, the lyophilized powder was dissolved in 1 mL of acetonitrile. In the case of NLCs, the EE% was determined by dilution of 500 μL of suspension of NPs with 500 μL of acetonitrile. The supernatant was then filtered using a hydrophilic syringe filter (pore of 0.22 μm) (Labfil, ALWSCI Corporation, Shaoxing, China) and injected in the UHPLC system. The concentration of EDV in each nanoformulation was determined by correlation with a calibration curve (Figure S3, Supporting Information). The EE% was calculated as the ratio between the amount of drug encapsulated within the NPs and the initial amount of drug used for NP synthesis as described previously [34].

### 3.12. Evaluation of Cytotoxicity of EDV and EDV-Loaded Nanoformulations

#### 3.12.1. Cell Culture Conditions

The evaluation of the cytotoxic effects of EDV and both loaded and unloaded nanoformulations was performed using a human neuroblastoma cell line (SH-SY5Y). Then, SH-SY5Y (ATCC^®^, HB-8065TM) cells were routinely cultured in 75 cm^2^ flasks using DMEM with 13.5 g/L glucose, supplemented with 10% heat-inactivated FBS (*v*/*v*), 1% NEAA (*v*/*v*), and 1% penicillin/streptomycin (*v*/*v*). Cells (passage 16–23) were maintained at 37 °C in a humidified atmosphere of 95% air/5% CO_2_, and the medium was changed every 3 days. Cultures were passed weekly by trypsinization (0.05% trypsin). In all experiments, the cells were seeded at a density of 2.5 × 10^4^ cells/cm^2^ with medium supplemented with 0.1% of retinoic acid (*v*/*v*) to promote the differentiation of the cells into cholinergic cells.

#### 3.12.2. Cell Viability Studies

After the differentiation process, the SH-SY5Y cells were treated with free EDV (12.5, 25 and 50 µM) and the nanoformulations for 24 h. The loaded nanoformulations were tested considering the amount of EDV encapsulated to maintain the same concentration compared with the free drug. Unloaded nanoformulations were used with the same volume as the loaded nanoformulations. After the exposure to free and encapsulated EDV, the cell viability was determined by the measurement of metabolic and lysosomal activities and cell mass by using the resazurin reduction, neutral red uptake, and sulforhodamine B (SRB) assays [34,35], respectively. In the neutral red uptake assay, following a 24 h incubation period, the cell culture medium was removed, and the fresh medium containing 150 µL of the dye (at a concentration of 50 µg/mL) was added. The cells were then incubated at 37 °C in a humified 5% CO_2_−95% air atmosphere for 1 h. After incubation, the cell culture medium was discarded, and a mixture of absolute ethanol and distilled water (1:1) containing 5% acetic acid was added to extract the dye accumulated within the lysosomes of viable cells. The absorbance at 540 nm was measured using a microplate reader (Synergy HTX Multi-Mode Reader; BioTek, Winooski, VT, USA). For the resazurin reduction assay, after 24 h of incubation time, the cell culture medium was removed, and a fresh medium supplemented with 100 µL of resazurin (10 µg/mL) was added. The plate was incubated for 45 min at 37 °C in a humidified 5% CO_2_−95% air atmosphere. Subsequently, the fluorescence signal was measured using a microplate reader (Synergy HTX MultiMode Reader; BioTek, Winooski, VT, USA), using an excitation wavelength of 540 nm and an emission wavelength of 590 nm. The cell culture medium was then removed, and the cells were fixed with a methanolic solution of acetic acid (1%) and left overnight at −20 °C. The next day, the medium was replaced with a 0.05% SRB solution, and the cells were incubated for 45 min at 37 °C in a humidified 5% CO_2_−95% air atmosphere. After incubation, the cells were washed with 1% acetic acid in water, and a Trizma solution (10 mM, pH 10.5) was added. The plate was stirred until the dye was completely dissolved. Finally, the absorbance at 510 nm was measured in a microplate reader (Synergy HTX Multimode Reader; BioTek, Winooski, VT, USA). For all assays, the results are presented as a percentage of the control (nontreated cells), taken as 100%. The results are presented as means ± standard error mean (SEM) of at least three independent experiments.

### 3.13. Measurement of Intracellular Oxidative Stress of EDV-Loaded Nanoformulations

The intracellular oxidative stress was measured by incubating differentiated SH-SY5Y cells with nonfluorescent probe 2′,7′-dichlorofluorescein diacetate (DCFH-DA) [47]. The probe DCFH-DA rapidly diffuses into cell membranes to produce 2′,7′-dichlorodihydrofluorescein (DCFH), which is oxidized by intracellular reactive oxygen and nitrogen species to the highly fluorescent 2′,7′-dichlorofluorescein (DCF) [47]. Differentiated SH-SY5Y cells were, firstly, pre-incubated with DCFH-DA (final concentration of 20 µM) for 90 min, at 37 °C, in a humidified 5% CO_2_–95% air atmosphere in the dark. At the end of the incubation period, the cell culture medium was replaced with a fresh culture medium containing the test compounds at 10 and 50 µM, and cells were incubated for 24 h. The fluorescence was then quantified in a microplate reader (Synergy HTX Multi-Mode Reader; BioTek, Winooski, VT, USA) set at 485 nm excitation and 530 nm emission. The results are expressed as the mean DCF fluorescence using cells treated with DCFH-DA as control cells. The results are presented as means ± standard error mean (SEM) of at least three independent experiments.

## 4. Statistical Analysis

The significant differences in collected data were analyzed by GraphPad Prism version 8.0.2. Two-way analysis of variance (2-way ANOVA) was used for statistical analysis, followed by a post hoc test (Dunnett’s test) for multiple comparison tests between groups. Differences were considered statistically significant at * *p* < 0.05, ** *p* < 0.01, *** *p* < 0.001, or **** *p* < 0.0001. In cellular studies, we established the percentage of 70% as a threshold to determine the cell viability of treated cells according to ISO 10993-5 [13].

## 5. Conclusions

Overcoming EDV’s poor solubility and stability, as well as its inability to cross the BBB, is an emerging challenge in the pursuit of the treatment of ALS. Using NPs as drug delivery systems to overcome these issues is a promising strategy in drug discovery. In this pioneering study, EDV was successfully encapsulated in PNPs, LPHNPs, and NLC. This study reveals some interesting insights into the differences between nanostructures regarding their size, PDI, zeta potential, and EE% as well as their cytotoxic outline. Overall, the usage of PEG or TPGS leads to a small decrease in size, for the PNPs and HNPs, as well as a decrease in the negative charge. When using higher pH values, the size also decreased while the PDI and EE% increased, although EDV was not stable. Overall, the nanoformulations tested did not show cytotoxicity in the SH-SY5Y cell line for the exception of the NLC formulations that showed some cytotoxicity possibly due to lipidic peroxidation. We highlighted the performance of Hyb-PLGA-TPGS@EDV NPs in terms of stability, EDV loading, and cell safety due to their antioxidant activity in the ABTS assay. This work reveals that the usage of NPs to overcome EDV’s drawbacks in the treatment of ALS is a promising strategy.

## Data Availability

No new data were created or analyzed in this study. Data sharing is not applicable to this article.

## Supplementary Materials

The following supporting information can be downloaded at: https://www.mdpi.com/article/10.3390/ijms26052146/s1.

## Abbreviations

Albumin nanoparticles (ANPs), lipid–polymer hybrid nanoparticles (LPHNPs), polymeric nanoparticles (PNPs), nanoparticles (NPs), nanostructured lipid carriers (NLC), edaravone (EDV).
