# Supplementary material for: Exploring Nano-Delivery Systems to Enhance the Edaravone Performance in Amyotrophic Lateral Sclerosis Treatment
Source: Int J Mol Sci. 2025 Feb 27;26(5):2146. doi: 10.3390/ijms26052146 (PMC11900301; doi:10.3390/ijms26052146)
Supplement: Supplementary file 1 [file ijms-26-02146-s001.zip › ijms-3468525-supplementary.pdf]

Supplementary Materials

Table of Contents

List of Tables

Table S1. The calibration set of compounds for the chromatographic hydrophobicity index (CHI) lipophilicity determination using pH 2.6 and 7.4.
Table S2. The calibration set of compounds for the CHI on Immobilized Artificial Membrane (IAM) determination using pH 7.4.
Table S3. Determination of the solubility of EDV in ultrapure water and in three different lipidic-based co-solvents and the parameters related with CHI and CHI(IAM).

List of Figures

Figure S1. Evaluation of the effect of the initial EDV feeding. The morphology was measured by D_{DLS} (A), PdI (B) and ZP values (C) of NLCs without and with EDV (10%, w/w lipid). The percentage of encapsulation efficiency (D, EE%) was determined using UHPLC technique.
Figure S2. D_{DLS} (A, B) and ZP values (C, D) of PNPs, LPHNPs and NLCs at different timepoints (days) under storage conditions at 25 °C (A, C) and 4 °C (B, D).
Figure S3. Cytotoxic effects of PLGA, Hyb-PLGA-TPGS and NLC in SH-SY5Y cells (12.5. 25 and 50 μM) after 24 h of exposure, by measuring the metabolic activity (A), lysosomal activity (B) and cell mass (C) by resazurin reduction method, neutral rd uptake and SRB assays, respectively. Intracellular ROS levels after exposure of SH-SY5Y cells (D) were also measured after 24 h of exposure with empty nanoformulations.
Figure S4. Radical scavenging activity of Edaravone as well as empty and loaded nanoformulations against ABTS•+ radical.

Evaluation of Physicochemical Properties of Edaravone

Table S1. Determination of the solubility of EDV in ultrapure water and in three different lipidic-based co-solvents and the parameters related with CHI and CHI(IAM).

**Solubility**		**Biomimetic Parameters**				
**Solvent**	**Concentration EDV (mg/g)**	**CHI LogD$_{7.4}$**	**CHI LogD$_{2.6}$**	**CHI(IAM)**	**Log K (IAM)**	**Kpcell**
**Milli-Q water**	0.165 ± 0.005	0.754	1.007	14.42	1.34	0.20
**Transcutol HP**	5.55 ± 0.73					
**Capryol™ 90**	5.33 ± 1.08					
**Capryol™ PGMC**	2.60 ± 0.12					

Table S2. The calibration set of compounds for the chromatographic hydrophobicity index (CHI) lipophilicity determination using pH 2.6 and 7.4.

	**pH 2.6**		pH 7.4	
**Reference compounds**	**CHI$_0$**	**t_r (min)**	**CHI$_0$**	**t_r (min)**
**Benzimidazole**	6.30	6.252	18.19	4.462
**Theophylline**	17.90	7.202	20.59	4.587
**Paracetamol**	18.77	7.311	24.12	4.741
**Caffeine**	23.41	7.487	30.71	5.116
**Colchicine**	43.90	8.342	44.32	5.582
**Carbamazepine**	60.42	9.289	58.45	6.297
**Indole**	72.10	10.189	69.15	6.905
**Propiophenone**	77.40	10.557	78.41	7.246
**Butyrophenone**	87.30	11.252	88.49	7.707
**Valerophenone**	96.40	11.972	97.67	8.110
**Heptanophenone**	112.10	13.619	111.80	8.816

Data are means from three independent experiments (n = 3).

Table S3. The calibration set of compounds for the CHI on Immobilized Artificial Membrane (IAM) determination using pH 7.4.

	pH 7.4	
**Reference compounds**	**CHI (IAM)$_0$**	**t_r (min)**
**Paracetamol**	2.90	2.655
**Acetanilide**	11.57	3.519
**Acetophenone**	17.07	3.966
**Propiophenone**	26.39	4.684
**Butyrophenone**	32.55	5.238
**Valerophenone**	37.71	5.704
**Hexanophenone**	41.91	6.095
**Heptanophenone**	45.49	6.422
**Octanophenone**	49.40	6.710

Data are means from three independent experiments (n = 3).

Edaravone Calibration Curve

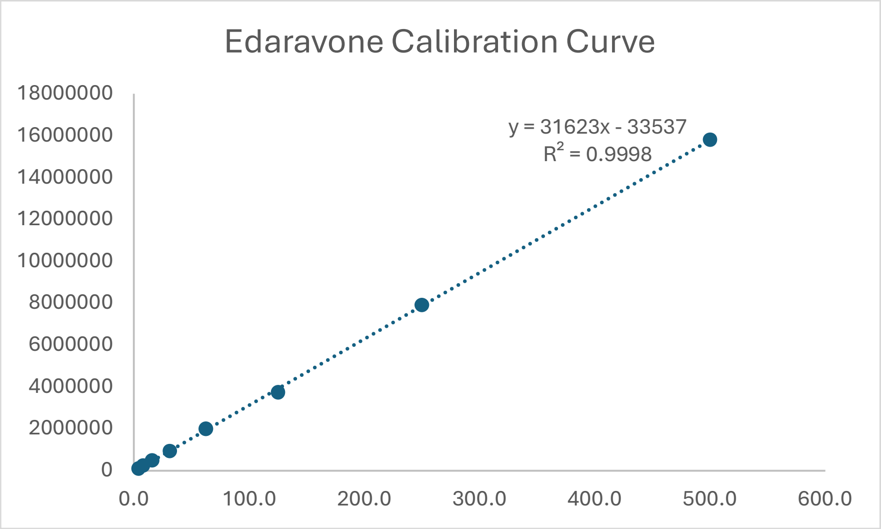

Edaravone calibration curve determined by UHPLC system with a mobile phase based on a mixture of water with 0.1% TFA and methanol (65:35).

Characterization of Nanoformulations Regarding Morphological and Physicochemical Features

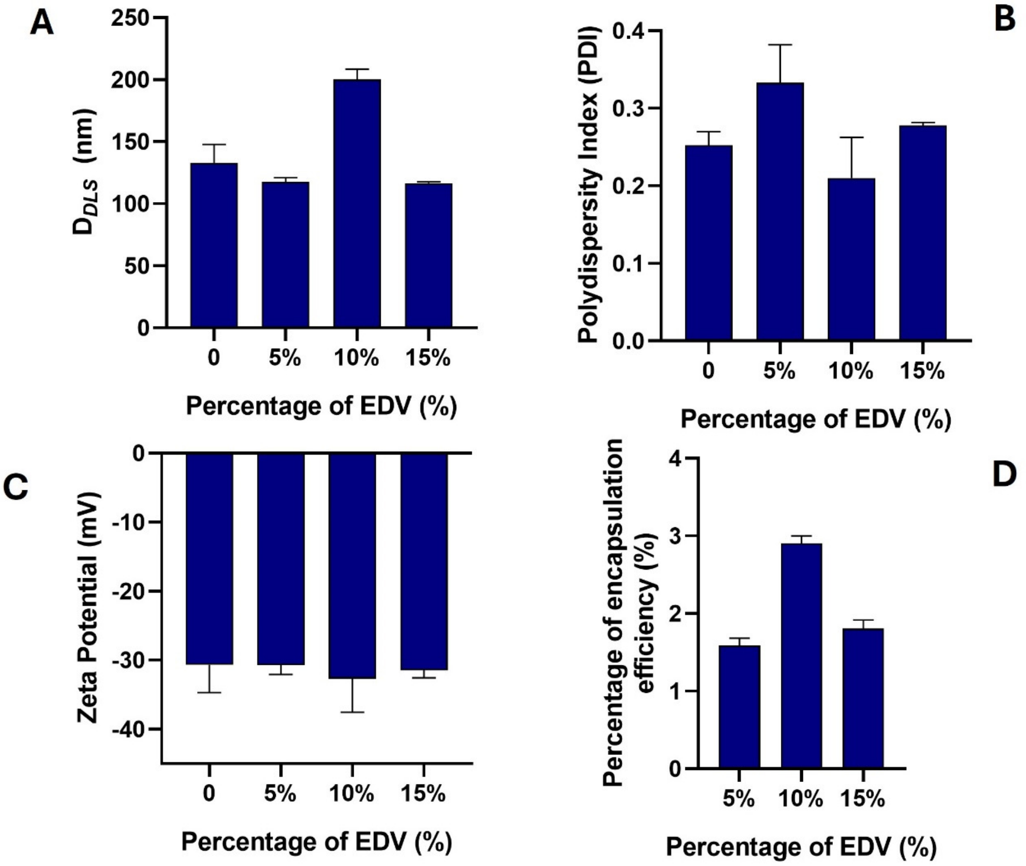

Figure S1. Evaluation of the effect of the initial EDV feeding. The morphology was measured by D_{DLS} (A), PdI (B) and ZP values (C) of NLCs without and with EDV (10%, w/w lipid). The percentage of encapsulation efficiency (D, EE%) was determined using UHPLC technique. Values are presented as mean ± SD of at least three independent syntheses of each nanoformulation.

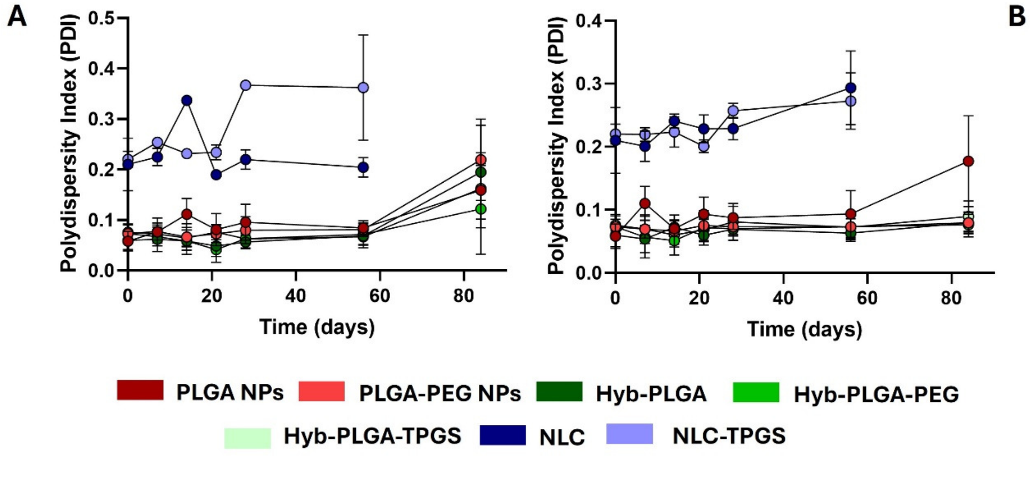

Figure S2. D_{DLS} (A, B) and ZP values (C, D) of PNPs, LPHNPs and NLCs at different timepoints (days) under storage conditions at 25 °C (A, C) and 4 °C (B, D). Values are presented as mean ± SD of at least three independent experiments.

Cellular Studies

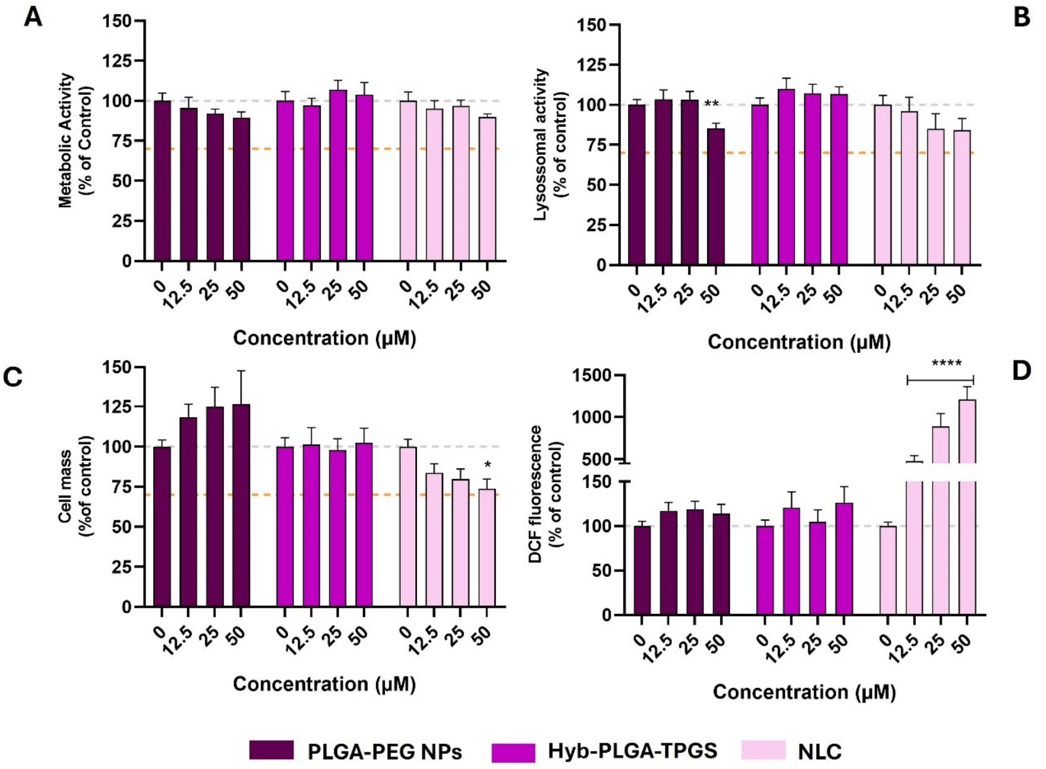

Figure S3. Cytotoxic effects of PLGA, Hyb-PLGA-TPGS and NLC in SH-SY5Y cells (12.5. 25 and 50 μM) after 24 h of exposure, by measuring the metabolic activity (A), lysosomal activity (B) and cell mass (C) by resazurin reduction method, neutral red uptake and SRB assays, respectively. Intracellular ROS levels after exposure of SH-SY5Y cells (D) were also measured after 24 h of exposure with empty nanoformulations. The data are expressed as the means of at least four independent experiments together with the standard error mean (mean ± SEM). Statistical comparisons were made using two-way ANOVA. In all cases, p values lower than 0.05 were considered significant (*$p < 0.05$, **$p < 0.01$, ****$p < 0.0001$ vs the control data; The grey dot line represents the mean of control cells (100 %) and the orange dashed line represents the cell viability limit of 70% (ISO 10993-5). .

Non-Cellular Studies

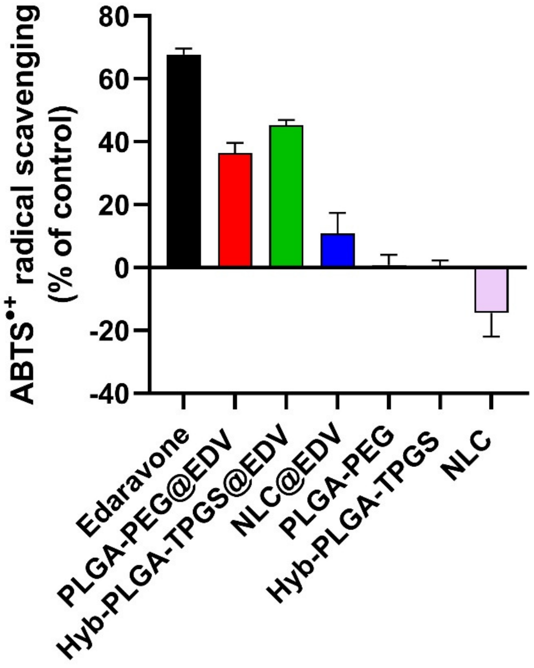

Figure S4. Radical scavenging activity of Edaravone as well as empty and loaded nanoformulations against ABTS•+ radical. The data are expressed as the means of three independent experiments together with the standard deviation (mean IC50 ± SD).
